# Drug and Opioid-Involved Overdose Deaths — United States, 2017–2018

**DOI:** 10.15585/mmwr.mm6911a4

**Published:** 2020-03-20

**Authors:** Nana Wilson, Mbabazi Kariisa, Puja Seth, Herschel Smith, Nicole L. Davis

**Affiliations:** ^1^Division of Overdose Prevention, National Center for Injury Prevention and Control, CDC; ^2^Oak Ridge Institute for Science and Education, Oak Ridge, Tennessee.

Of the 70,237 drug overdose deaths in the United States in 2017, approximately two thirds (47,600) involved an opioid (*1*). In recent years, increases in opioid-involved overdose deaths have been driven primarily by deaths involving synthetic opioids other than methadone (hereafter referred to as synthetic opioids) (*1*). CDC analyzed changes in age-adjusted death rates from 2017 to 2018 involving all opioids and opioid subcategories* by demographic characteristics, county urbanization levels, U.S. Census region, and state. During 2018, a total of 67,367 drug overdose deaths occurred in the United States, a 4.1% decline from 2017; 46,802 (69.5%) involved an opioid (*2*). From 2017 to 2018, deaths involving all opioids, prescription opioids, and heroin decreased 2%, 13.5%, and 4.1%, respectively. However, deaths involving synthetic opioids increased 10%, likely driven by illicitly manufactured fentanyl (IMF), including fentanyl analogs (*1*,*3*). Efforts related to all opioids, particularly deaths involving synthetic opioids, should be strengthened to sustain and accelerate declines in opioid-involved deaths. Comprehensive surveillance and prevention measures are critical to reducing opioid-involved deaths, including continued surveillance of evolving drug use and overdose, polysubstance use, and the changing illicit drug market; naloxone distribution and outreach to groups at risk for IMF exposure; linkage to evidence-based treatment for persons with substance use disorders; and continued partnerships with public safety.

Drug overdose deaths were identified in National Vital Statistics System multiple cause-of-death mortality files^†^ using the *International Classification of Diseases, Tenth Revision* (ICD-10) underlying cause-of-death codes X40–X44 (unintentional), X60–X64 (suicide), X85 (homicide), or Y10–Y14 (undetermined intent). Among deaths with drug overdose as the underlying cause, the opioid subcategory was determined by the following ICD-10 multiple cause-of-death codes: all opioids (T40.0, T40.1, T40.2, T40.3, T40.4, or T40.6)^§^; prescription opioids (T40.2 or T40.3); heroin (T40.1); and synthetic opioids other than methadone (T40.4). Some deaths involved more than one opioid subcategory and were included in the rates for each; subcategories are not mutually exclusive.^¶^

Changes from 2017 to 2018 in age-adjusted overdose death rates** were examined for all opioids, prescription opioids, heroin, and synthetic opioids. Death rates were stratified by age, sex, race/ethnicity, urbanization level,^††^ U.S. Census region,^§§^ and state. State-level analyses included 38 states and the District of Columbia (DC) with adequate drug specificity^¶¶^ for 2017 and 2018.*** The drug or drugs involved in the drug overdose death were not specified on 12% of drug overdose death certificates in 2017 and on 8% of those from 2018. The percentage of 2018 death certificates with at least one drug specified ranged from 54.1% to 100% among states. Changes in death rates from 2017 to 2018 were compared using z-tests when deaths were ≥100 and nonoverlapping confidence intervals based on a gamma distribution when <100.^†††^ Changes presented in the text represent statistically significant findings, unless otherwise specified.

During 2018, drug overdoses resulted in 67,367 deaths in the United States, a 4.1% decrease from 2017. Among these drug overdose deaths, 46,802 (69.5%) involved an opioid. From 2017 to 2018, opioid-involved death rates decreased 2.0%, from 14.9 per 100,000 population to 14.6 (Table 1); decreases occurred among females; persons aged 15–34 years and 45–54 years; non-Hispanic whites; and in small metro, micropolitan, and noncore areas; and in the Midwest and South regions. Rates during 2017–2018 increased among persons aged ≥65 years, non-Hispanic blacks, and Hispanics, and in the Northeast and the West regions. Rates decreased in 11 states and DC and increased in three states, with the largest relative (percentage) decrease in Iowa (–30.4%) and the largest absolute decrease (difference in rates) in Ohio (–9.6); the largest relative and absolute increase occurred in Missouri (18.8%, 3.1). The highest opioid-involved death rate in 2018 was in West Virginia (42.4 per 100,000).

**TABLE 1:** Annual number and age-adjusted rate of drug overdose deaths* involving all opioids^†^ and prescription opioids,^§^^,^^¶^ by sex, age, race/ethnicity,** urbanization level,^††^ U.S. Census region,^§§^ and selected states^¶¶^ — National Vital Statistics System, United States, 2017 and 2018

Decedent characteristic	All opioids				Prescription opioids			
	2017	2018	Rate change from 2017 to 2018***		2017	2018	Rate change from 2017 to 2018***	
	No. (rate)	No. (rate)	Absolute change	Relative change	No. (rate)	No. (rate)	Absolute change	Relative change
**All**	**47,600 (14.9)**	**46,802 (14.6)**	**−0.3^†††^**	**−2.0^†††^**	**17,029 (5.2)**	**14,975 (4.5)**	**−0.7^†††^**	**−13.5^†††^**
**Sex**								
Male	32,337 (20.4)	32,078 (20.1)	−0.3	−1.5	9,873 (6.1)	8,723 (5.3)	−0.8**^†††^**	−13.1**^†††^**
Female	15,263 (9.4)	14,724 (9.0)	−0.4**^†††^**	−4.3**^†††^**	7,156 (4.2)	6,252 (3.7)	−0.5**^†††^**	−11.9**^†††^**
**Age group (yrs)**								
0–14	79 (0.1)	65 (0.1)	0.0	0.0	50 (0.1)	36 (0.1)	0.0	0.0
15–24	4,094 (9.5)	3,618 (8.4)	−1.1**^†††^**	−11.6**^†††^**	1,050 (2.4)	790 (1.8)	−0.6**^†††^**	−25.0**^†††^**
25–34	13,181 (29.1)	12,839 (28.1)	−1.0**^†††^**	−3.4**^†††^**	3,408 (7.5)	2,862 (6.3)	−1.2**^†††^**	−16.0**^†††^**
35–44	11,149 (27.3)	11,414 (27.7)	0.4	1.5	3,714 (9.1)	3,350 (8.1)	−1.0**^†††^**	−11.0**^†††^**
45–54	10,207 (24.1)	9,565 (23.0)	−1.1**^†††^**	−4.6**^†††^**	4,238 (10.0)	3,490 (8.4)	−1.6**^†††^**	−16.0**^†††^**
55–64	7,153 (17.0)	7,278 (17.2)	0.2	1.2	3,509 (8.4)	3,291 (7.8)	−0.6**^†††^**	−7.1**^†††^**
≥65	1,724 (3.4)	2,012 (3.8)	0.4**^†††^**	11.8**^†††^**	1,055 (2.1)	1,152 (2.2)	0.1	4.8
**Sex and age group (yrs)**								
Male 15–24	2,885 (13.0)	2,527 (11.5)	−1.5**^†††^**	−11.5**^†††^**	728 (3.3)	548 (2.5)	−0.8**^†††^**	−24.2**^†††^**
Male 25–44	17,352 (40.0)	17,240 (39.4)	−0.6	−1.5	4,516 (10.4)	3,895 (8.9)	−1.5**^†††^**	−14.4**^†††^**
Male 45–64	11,061 (26.9)	10,986 (26.8)	−0.1	−0.4	4,089 (9.9)	3,637 (8.9)	−1.0**^†††^**	−10.1**^†††^**
Female 15–24	1,209 (5.7)	1,091 (5.2)	−0.5**^†††^**	−8.8**^†††^**	322 (1.5)	242 (1.2)	−0.3**^†††^**	−20.0**^†††^**
Female 25–44	6,978 (16.3)	7,013 (16.2)	−0.1	−0.6	2,606 (6.1)	2,317 (5.4)	−0.7**^†††^**	−11.5**^†††^**
Female 45–64	6,299 (14.6)	5,857 (13.6)	−1.0**^†††^**	−6.8**^†††^**	3,658 (8.5)	3,144 (7.3)	−1.2**^†††^**	−14.1**^†††^**
Race/Ethnicity**								
White, non-Hispanic	37,113 (19.4)	35,363 (18.6)	−0.8**^†††^**	−4.1**^†††^**	13,900 (6.9)	12,085 (6.0)	−0.9**^†††^**	−13.0**^†††^**
Black, non-Hispanic	5,513 (12.9)	6,088 (14.0)	1.1**^†††^**	8.5**^†††^**	1,508 (3.5)	1,444 (3.3)	−0.2	−5.7
Hispanic	3,932 (6.8)	4,370 (7.5)	0.7**^†††^**	10.3**^†††^**	1,211 (2.2)	1,122 (2.0)	−0.2**^†††^**	−9.1**^†††^**
American Indian/Alaska Native, non-Hispanic	408 (15.7)	373 (14.2)	−1.5	−9.6	187 (7.2)	125 (4.7)	−2.5**^†††^**	−34.7**^†††^**
Asian/Pacific Islander, non-Hispanic	348 (1.6)	345 (1.5)	−0.1	−6.3	130 (0.6)	115 (0.5)	−0.1	−16.7
**County urbanization level^††^**								
Large central metro	14,518 (13.9)	14,767 (14.1)	0.2	1.4	4,945 (4.7)	4,394 (4.1)	−0.6**^†††^**	−12.8**^†††^**
Large fringe metro	13,594 (17.2)	13,476 (17.0)	−0.2	−1.2	4,273 (5.2)	3,791 (4.6)	−0.6**^†††^**	−11.5**^†††^**
Medium metro	10,561 (16.2)	10,328 (15.8)	−0.4	−2.5	3,951 (5.9)	3,539 (5.2)	−0.7**^†††^**	−11.9**^†††^**
Small metro	3,560 (12.9)	3,379 (12.2)	−0.7**^†††^**	−5.4**^†††^**	1,479 (5.2)	1,278 (4.5)	−0.7**^†††^**	−13.5**^†††^**
Micropolitan (nonmetro)	3,462 (13.9)	3,162 (12.7)	−1.2**^†††^**	−8.6**^†††^**	1,440 (5.6)	1,240 (4.7)	−0.9**^†††^**	−16.1**^†††^**
Noncore (nonmetro)	1,905 (11.2)	1,690 (10.1)	−1.1**^†††^**	−9.8**^†††^**	941 (5.3)	733 (4.1)	−1.2**^†††^**	−22.6**^†††^**
**U.S. Census region of residence^§§^**								
Northeast	11,784 (21.3)	12,467 (22.8)	1.5**^†††^**	7.0**^†††^**	3,047 (5.3)	2,991 (5.3)	0.0	0.0
Midwest	12,483 (19.1)	11,268 (17.2)	−1.9**^†††^**	−9.9**^†††^**	3,702 (5.5)	2,965 (4.4)	−1.1**^†††^**	−20.0**^†††^**
South	16,999 (14.1)	16,413 (13.5)	−0.6**^†††^**	−4.3**^†††^**	6,929 (5.6)	5,936 (4.7)	−0.9**^†††^**	−16.1**^†††^**
West	6,334 (8.0)	6,654 (8.3)	0.3**^†††^**	3.8**^†††^**	3,351 (4.1)	3,083 (3.8)	−0.3**^†††^**	−7.3**^†††^**
**States with very good to excellent reporting (n = 29)^¶¶^**								
Alaska	102 (13.9)	68 (8.8)	−5.1	−36.7	51 (7.0)	38 (4.9)	−2.1	−30.0
Arizona	928 (13.5)	1,106 (15.9)	2.4**^†††^**	17.8**^†††^**	414 (5.9)	362 (5.0)	−0.9**^†††^**	−15.3**^†††^**
Connecticut	955 (27.7)	948 (27.5)	−0.2	−0.7	273 (7.7)	231 (6.4)	−1.3	−16.9
District of Columbia	244 (34.7)	191 (26.7)	−8.0**^†††^**	−23.1**^†††^**	58 (8.4)	41 (5.7)	−2.7	−32.1
Georgia	1,014 (9.7)	866 (8.3)	−1.4**^†††^**	−14.4**^†††^**	568 (5.4)	440 (4.1)	−1.3**^†††^**	−24.1**^†††^**
Illinois	2,202 (17.2)	2,169 (17.0)	−0.2	−1.2	623 (4.8)	539 (4.2)	−0.6**^†††^**	−12.5**^†††^**
Iowa	206 (6.9)	143 (4.8)	−2.1**^†††^**	−30.4**^†††^**	104 (3.4)	64 (2.1)	−1.3**^†††^**	−38.2**^†††^**
Maine	360 (29.9)	282 (23.4)	−6.5**^†††^**	−21.7**^†††^**	100 (7.6)	69 (5.1)	−2.5	−32.9
Maryland	1,985 (32.2)	2,087 (33.7)	1.5	4.7	711 (11.5)	576 (9.2)	−2.3**^†††^**	−20.0**^†††^**
Massachusetts	1,913 (28.2)	1,991 (29.3)	1.1	3.9	321 (4.6)	331 (4.7)	0.1	2.2
Missouri	952 (16.5)	1,132 (19.6)	3.1**^†††^**	18.8**^†††^**	253 (4.1)	265 (4.4)	0.3	7.3
Nevada	412 (13.3)	372 (11.5)	−1.8	−13.5	276 (8.7)	235 (7.2)	−1.5**^†††^**	−17.2**^†††^**
New Hampshire	424 (34.0)	412 (33.1)	−0.9	−2.6	62 (4.8)	43 (3.1)	−1.7	−35.4
New Mexico	332 (16.7)	338 (16.7)	0.0	0.0	171 (8.5)	176 (8.2)	−0.3	−3.5
New York	3,224 (16.1)	2,991 (15.1)	−1.0**^†††^**	−6.2**^†††^**	1,044 (5.1)	998 (4.9)	−0.2	−3.9
North Carolina	1,953 (19.8)	1,783 (17.9)	−1.9**^†††^**	−9.6**^†††^**	659 (6.5)	489 (4.7)	−1.8**^†††^**	−27.7**^†††^**
Ohio	4,293 (39.2)	3,237 (29.6)	−9.6**^†††^**	−24.5**^†††^**	947 (8.4)	571 (5.0)	−3.4**^†††^**	−40.5**^†††^**
Oklahoma	388 (10.2)	308 (7.8)	−2.4**^†††^**	−23.5**^†††^**	251 (6.7	172 (4.3)	−2.4**^†††^**	−35.8**^†††^**
Oregon	344 (8.1)	339 (8.0)	−0.1	−1.2	154 (3.5)	151 (3.4)	−0.1	−2.9
Rhode Island	277 (26.9)	267 (25.9)	−1.0	−3.7	99 (8.8)	85 (7.7)	−1.1	−12.5
South Carolina	749 (15.5)	835 (17.1)	1.6	10.3	345 (7.1)	375 (7.4)	0.3	4.2
Tennessee	1,269 (19.3)	1,307 (19.9)	0.6	3.1	644 (9.6)	550 (8.2)	−1.4**^†††^**	−14.6**^†††^**
Utah	456 (15.5)	437 (14.8)	−0.7	−4.5	315 (10.8)	306 (10.5)	−0.3	−2.8
Vermont	114 (20.0)	127 (22.8)	2.8	14.0	40 (6.3)	27 (4.4)	−1.9	−30.2
Virginia	1,241 (14.8)	1,193 (14.3)	−0.5	−3.4	404 (4.7)	326 (3.8)	−0.9**^†††^**	−19.1**^†††^**
Washington	742 (9.6)	737 (9.4)	−0.2	−2.1	343 (4.3)	301 (3.8)	−0.5	−11.6
West Virginia	833 (49.6)	702 (42.4)	−7.2**^†††^**	−14.5**^†††^**	304 (17.2)	234 (13.1)	−4.1**^†††^**	−23.8**^†††^**
Wisconsin	926 (16.9)	846 (15.3)	−1.6**^†††^**	−9.5**^†††^**	362 (6.4)	301 (5.3)	−1.1**^†††^**	−17.2**^†††^**
Wyoming	47 (8.7)	40 (6.8)	−1.9	−21.8	31 (6.0)	28 (4.6)	−1.4	−23.3
**States with good reporting (n = 10)^¶¶^**								
California	2,199 (5.3)	2,410 (5.8)	0.5**^†††^**	9.4**^†††^**	1,169 (2.8)	1,084 (2.6)	−0.2	−7.1
Colorado	578 (10.0)	564 (9.5)	−0.5	−5.0	300 (5.1)	268 (4.4)	−0.7	−13.7
Florida	3,245 (16.3)	3,189 (15.8)	−0.5	−3.1	1,272 (6.0)	1,282 (6.0)	0.0	0.0
Hawaii	53 (3.4)	59 (4.1)	0.7	20.6	40 (2.5)	33 (2.3)	−0.2	−8.0
Indiana	1,176 (18.8)	1,104 (17.5)	−1.3	−6.9	425 (6.6)	370 (5.6)	−1.0**^†††^**	−15.2**^†††^**
Kentucky	1,160 (27.9)	989 (23.4)	−4.5**^†††^**	−16.1**^†††^**	433 (10.2)	315 (7.2)	−3.0**^†††^**	−29.4**^†††^**
Michigan	2,033 (21.2)	2,011 (20.8)	−0.4	−1.9	633 (6.5)	556 (5.6)	−0.9**^†††^**	−13.8**^†††^**
Minnesota	422 (7.8)	343 (6.3)	−1.5**^†††^**	−19.2**^†††^**	195 (3.6)	136 (2.5)	−1.1**^†††^**	−30.6**^†††^**
Mississippi	185 (6.4)	173 (6.1)	−0.3	−4.7	96 (3.2)	92 (3.1)	−0.1	−3.1
Texas	1,458 (5.1)	1,402 (4.8)	−0.3	−5.9	646 (2.3)	547 (1.9)	−0.4	−17.4

* Deaths were classified using *the International Classification of Diseases, Tenth Revision* (ICD–10). Drug overdose deaths were identified using underlying cause-of-death codes X40–X44, X60–X64, X85, and Y10–Y14. Rates are age-adjusted using the direct method and the 2000 U.S. standard population, except for age-specific crude rates. All rates are per 100,000 population.

^†^ Drug overdose deaths, as defined, that have opium (T40.0), heroin (T40.1), natural and semisynthetic opioids (T40.2), methadone (T40.3), synthetic opioids other than methadone (T40.4) or other and unspecified narcotics (T40.6) as a contributing cause.

^§^ Drug overdose deaths, as defined, that have natural and semisynthetic opioids (T40.2) or methadone (T40.3) as a contributing cause.

^¶^ Categories of deaths are not exclusive as deaths might involve more than one drug category. Summing of categories will result in more than the total number of deaths in a year.

** Data for Hispanic origin should be interpreted with caution; studies comparing Hispanic origin on death certificates and on Census surveys have shown inconsistent reporting on Hispanic ethnicity. Potential race misclassification might lead to underestimates for certain categories, primarily American Indian/Alaska Native non-Hispanic and Asian/Pacific Islander non-Hispanic decedents. https://www.cdc.gov/nchs/data/series/sr_02/sr02_172.pdf.

^††^ By the 2013 National Center for Health Statistics Urban-Rural Classification Scheme for Counties. https://www.cdc.gov/nchs/data_access/urban_rural.htm.

^§§^ *Northeast*: Connecticut, Maine, Massachusetts, New Hampshire, New Jersey, New York, Pennsylvania, Rhode Island, and Vermont. *Midwest*: Illinois, Indiana, Iowa, Kansas, Michigan, Minnesota, Missouri, Nebraska, North Dakota, Ohio, South Dakota, and Wisconsin. *South*: Alabama, Arkansas, Delaware, District of Columbia, Florida, Georgia, Kentucky, Louisiana, Maryland, Mississippi, North Carolina, Oklahoma, South Carolina, Tennessee, Texas, Virginia, and West Virginia. *West*: Alaska, Arizona, California, Colorado, Hawaii, Idaho, Montana, Nevada, New Mexico, Oregon, Utah, Washington, and Wyoming.

^¶¶^ Analyses were limited to states meeting the following criteria. States with very good to excellent reporting had ≥90% of drug overdose deaths mention at least one specific drug in 2017, with the change in drug overdose deaths mentioning of at least one specific drug differing by <10 percentage points from 2017 to 2018. States with good reporting had 80% to <90% of drug overdose deaths mention at least one specific drug in 2017, with the change in the percentage of drug overdose deaths mentioning at least one specific drug differing by <10 percentage points from 2017 to 2018. States included also were required to have stable rate estimates (i.e., based on ≥20 deaths in at least two of the following drug categories: opioids, prescription opioids, synthetic opioids other than methadone, and heroin).

*** Absolute rate change is the difference between 2017 and 2018 rates. Relative rate change is the absolute rate change divided by the 2017 rate, multiplied by 100. Nonoverlapping confidence intervals based on the gamma method were used if the number of deaths was <100 in 2017 or 2018, and z-tests were used if the number of deaths was ≥100 in both 2017 and 2018.

^†††^ Statistically significant (p-value <0.05).

Prescription opioid-involved death rates decreased by 13.5% from 2017 to 2018. Rates decreased in males and females, persons aged 15–64 years, non-Hispanic whites, Hispanics, non-Hispanic American Indian/Alaska Natives, and across all urbanization levels. Prescription opioid–involved death rates remained stable in the Northeast and decreased in the Midwest, South, and the West. Seventeen states experienced declines in prescription opioid–involved death rates, with no states experiencing significant increases. The largest relative decrease occurred in Ohio (–40.5%), whereas the largest absolute decrease occurred in West Virginia (–4.1), which also had the highest prescription opioid-involved death rate in 2018 (13.1 per 100,000).

Heroin-involved death rates decreased 4.1% from 2017 to 2018; reductions occurred among males and females, persons aged 15–34 years, non-Hispanic whites, and in large central metro and large fringe metro areas (Table 2). Rates decreased in the Midwest and increased in the West. Rates decreased in seven states and DC and increased in three states from 2017 to 2018. The largest relative decrease occurred in Kentucky (50.0%), and the largest absolute decrease occurred in DC (–7.1); the largest relative and absolute increase was in Tennessee (18.8%, 0.9). The highest heroin-involved death rate in 2018 was in Vermont (12.5 per 100,000).

**TABLE 2:** Annual number and age-adjusted rate of drug overdose deaths* involving heroin^†^ and synthetic opioids other than methadone,^§^^,^^¶^ by sex, age, race/ethnicity,** urbanization level,^††^ U.S. Census region,^§§^ and selected states^¶¶^ — National Vital Statistics System, United States, 2017 and 2018

Decedent characteristic	Heroin				Synthetic opioids other than methadone			
	2017	2018	Rate change from 2017 to 2018***		2017	2018	Rate change from 2017 to 2018***	
	No. (rate)	No. (rate)	Absolute change	Relative change	No. (rate)	No. (rate)	Absolute change	Relative change
**All**	**15,482 (4.9)**	**14,996 (4.7)**	**−0.2^†††^**	**−4.1^†††^**	**28,466 (9.0)**	**31,335 (9.9)**	**0.9^†††^**	**10.0^†††^**
**Sex**								
Male	11,596 (7.3)	11,291 (7.1)	−0.2**^†††^**	−2.7**^†††^**	20,524 (13.0)	22,528 (14.2)	1.2**^†††^**	9.2**^†††^**
Female	3,886 (2.5)	3,705 (2.3)	−0.2**^†††^**	−8.0**^†††^**	7,942 (5.0)	8,807 (5.5)	0.5**^†††^**	10.0**^†††^**
**Age group (yrs)**								
0–14	—^§§§^	—^§§§^	—^§§§^	—^§§§^	33 (0.1)	29 (0.1)	0.0	0.0
15–24	1,454 (3.4)	1,160 (2.7)	−0.7**^†††^**	−20.6**^†††^**	2,655 (6.1)	2,640 (6.1)	0.0	0.0
25–34	4,890 (10.8)	4,642 (10.2)	−0.6**^†††^**	−5.6**^†††^**	8,825 (19.5)	9,568 (20.9)	1.4**^†††^**	7.2**^†††^**
35–44	3,713 (9.1)	3,740 (9.1)	0.0	0.0	7,084 (17.3)	8,070 (19.6)	2.3**^†††^**	13.3**^†††^**
45–54	3,043 (7.2)	2,922 (7.0)	−0.2	−2.8	5,762 (13.6)	6,132 (14.7)	1.1**^†††^**	8.1**^†††^**
55–64	2,005 (4.8)	2,077 (4.9)	0.1	2.1	3,481 (8.3)	4,018 (9.5)	1.2**^†††^**	14.5**^†††^**
≥65	368 (0.7)	445 (0.8)	0.1	14.3	620 (1.2)	871 (1.7)	0.5**^†††^**	41.7**^†††^**
**Sex and age group (yrs)**								
Male 15–24	1,031 (4.7)	821 (3.7)	−1.0**^†††^**	−21.3**^†††^**	1,877 (8.5)	1,841 (8.4)	−0.1	−1.2
Male 25–44	6,428 (14.8)	6,305 (14.4)	−0.4	−2.7	11,693 (27.0)	12,810 (29.2)	2.2**^†††^**	8.1**^†††^**
Male 45–64	3,830 (9.3)	3,778 (9.2)	−0.1	−1.1	6,524 (15.8)	7,195 (17.6)	1.8**^†††^**	11.4**^†††^**
Female 15–24	423 (2.0)	339 (1.6)	−0.4**^†††^**	−20.0**^†††^**	778 (3.7)	799 (3.8)	0.1	2.7
Female 25–44	2,175 (5.1)	2,077 (4.8)	−0.3	−5.9	4,216 (9.8)	4,828 (11.2)	1.4**^†††^**	14.3**^†††^**
Female 45–64	1,218 (2.8)	1,221 (2.8)	0.0	0.0	2,719 (6.3)	2,955 (6.9)	0.6**^†††^**	9.5**^†††^**
Race/Ethnicity**								
White, non-Hispanic	11,293 (6.1)	10,756 (5.8)	−0.3**^†††^**	−4.9**^†††^**	21,956 (11.9)	23,214 (12.6)	0.7**^†††^**	5.9**^†††^**
Black, non-Hispanic	2,140 (4.9)	2,145 (4.9)	0.0	0.0	3,832 (9.0)	4,780 (11.0)	2.0**^†††^**	22.2**^†††^**
Hispanic	1,669 (2.9)	1,768 (3.1)	0.2	6.9	2,152 (3.7)	2,766 (4.7)	1.0**^†††^**	27.0**^†††^**
American Indian/Alaska Native, non-Hispanic	136 (5.2)	133 (5.1)	−0.1	−1.9	171 (6.5)	191 (7.3)	0.8	12.3
Asian/Pacific Islander, non-Hispanic	119 (0.5)	85 (0.4)	−0.1	−20.0	189 (0.8)	214 (1.0)	0.2**^†††^**	25.0**^†††^**
**County urbanization level^††^**								
Large central metro	5,820 (5.6)	5,467 (5.2)	−0.4**^†††^**	−7.1**^†††^**	8,511 (8.2)	9,804 (9.4)	1.2**^†††^**	14.6**^†††^**
Large fringe metro	4,526 (5.8)	4,321 (5.5)	−0.3**^†††^**	−5.2**^†††^**	8,991 (11.6)	9,871 (12.7)	1.1**^†††^**	9.5**^†††^**
Medium metro	2,973 (4.6)	3,091 (4.8)	0.2	4.3	6,254 (9.8)	6,750 (10.5)	0.7**^†††^**	7.1**^†††^**
Small metro	972 (3.6)	949 (3.5)	−0.1	−2.8	1,878 (7.0)	2,050 (7.6)	0.6**^†††^**	8.6**^†††^**
Micropolitan (nonmetro)	801 (3.3)	780 (3.3)	0.0	0.0	1,860 (7.7)	1,925 (8.0)	0.3	3.9
Noncore (nonmetro)	390 (2.4)	388 (2.4)	0.0	0.0	972 (6.0)	935 (5.8)	−0.2	−3.3
**U.S. Census region of residence^§§^**								
Northeast	4,310 (7.8)	4,363 (8.0)	0.2	2.6	8,861 (16.2)	10,351 (19.1)	2.9**^†††^**	17.9**^†††^**
Midwest	4,228 (6.5)	3,575 (5.5)	−1.0**^†††^**	−15.4**^†††^**	8,234 (12.8)	8,348 (12.9)	0.1	0.8
South	4,776 (4.0)	4,718 (3.9)	−0.1	−2.5	9,906 (8.3)	10,443 (8.6)	0.3**^†††^**	3.6**^†††^**
West	2,168 (2.8)	2,340 (3.0)	0.2**^†††^**	7.1**^†††^**	1,465 (1.9)	2,193 (2.8)	0.9**^†††^**	47.4**^†††^**
**States with very good to excellent reporting (n = 29)^¶¶^**								
Alaska	36 (4.9)	29 (3.8)	−1.1	−22.4	37 (4.9)	18 –^§§§^	–^§§§^	–^§§§^
Arizona	334 (5.0)	352 (5.2)	0.2	4.0	267 (4.0)	522 (7.7)	3.7**^†††^**	92.5**^†††^**
Connecticut	425 (12.4)	338 (9.9)	−2.5**^†††^**	−20.2**^†††^**	686 (20.3)	767 (22.5)	2.2	10.8
District of Columbia	127 (18)	79 (10.9)	−7.1**^†††^**	−39.4**^†††^**	182 (25.7)	162 (22.6)	−3.1	−12.1
Georgia	263 (2.6)	299 (2.9)	0.3	11.5	419 (4.1)	349 (3.4)	−0.7**^†††^**	−17.1**^†††^**
Illinois	1,187 (9.2)	1,050 (8.3)	−0.9**^†††^**	−9.8**^†††^**	1,251 (9.8)	1,568 (12.4)	2.6**^†††^**	26.5**^†††^**
Iowa	61 (2.1)	37 (1.3)	−0.8	−38.1	92 (3.2)	80 (2.8)	−0.4	−12.5
Maine	76 (6.2)	71 (6.0)	−0.2	−3.2	278 (23.5)	229 (19.8)	−3.7	−15.7
Maryland	522 (8.6)	356 (5.9)	−2.7**^†††^**	−31.4**^†††^**	1,542 (25.2)	1,825 (29.6)	4.4**^†††^**	17.5**^†††^**
Massachusetts	466 (7.0)	475 (7.0)	0.0	0.0	1,649 (24.5)	1,806 (26.8)	2.3**^†††^**	9.4**^†††^**
Missouri	299 (5.3)	351 (6.1)	0.8	15.1	618 (10.9)	868 (15.3)	4.4**^†††^**	40.4**^†††^**
Nevada	94 (3.1)	108 (3.5)	0.4	12.9	66 (2.2)	85 (2.8)	0.6	27.3
New Hampshire	28 (2.4)	12 –^§§§^	–^§§§^	–^§§§^	374 (30.4)	386 (31.3)	0.9	3.0
New Mexico	144 (7.4)	130 (6.6)	−0.8	−10.8	75 (3.7)	105 (5.4)	1.7	45.9
New York	1,356 (6.8)	1,243 (6.3)	−0.5	−7.4	2,238 (11.3)	2,195 (11.2)	−0.1	−0.9
North Carolina	537 (5.6)	619 (6.3)	0.7	12.5	1,285 (13.2)	1,272 (13.0)	−0.2	−1.5
Ohio	1,000 (9.2)	721 (6.6)	−2.6**^†††^**	−28.3**^†††^**	3,523 (32.4)	2,783 (25.7)	−6.7**^†††^**	−20.7**^†††^**
Oklahoma	61 (1.6)	84 (2.2)	0.6	37.5	102 (2.6)	79 (2.0)	−0.6	−23.1
Oregon	124 (3.0)	154 (3.7)	0.7	23.3	85 (2.1)	97 (2.4)	0.3	14.3
Rhode Island	14—^§§§^	24 (2.2)	–^§§§^	–^§§§^	201 (20.1)	213 (21.0)	0.9	4.5
South Carolina	153 (3.2)	183 (3.8)	0.6	18.8	404 (8.5)	510 (10.8)	2.3**^†††^**	27.1**^†††^**
Tennessee	311 (4.8)	369 (5.7)	0.9**^†††^**	18.8**^†††^**	590 (9.3)	827 (12.8)	3.5**^†††^**	37.6**^†††^**
Utah	147 (4.8)	156 (5.1)	0.3	6.3	92 (3.1)	83 (2.9)	−0.2	−6.5
Vermont	41 (7.3)	68 (12.5)	5.2	71.2	77 (13.8)	106 (19.3)	5.5	39.9
Virginia	556 (6.7)	532 (6.4)	−0.3	−4.5	829 (10.0)	852 (10.3)	0.3	3.0
Washington	306 (4.0)	328 (4.2)	0.2	5.0	143 (1.9)	221 (2.9)	1.0**^†††^**	52.6**^†††^**
West Virginia	244 (14.9)	195 (12.3)	−2.6	−17.4	618 (37.4)	551 (34.0)	−3.4	−9.1
Wisconsin	414 (7.8)	327 (6.0)	−1.8**^†††^**	−23.1**^†††^**	466 (8.6)	506 (9.4)	0.8	9.3
Wyoming	—^§§§^	—^§§§^	—^§§§^	—^§§§^	—^§§§^	—^§§§^	—^§§§^	—^§§§^
**States with good reporting (n = 10)^¶¶^**								
California	715 (1.7)	778 (1.9)	0.2**^†††^**	11.8**^†††^**	536 (1.3)	865 (2.2)	0.9**^†††^**	69.2**^†††^**
Colorado	224 (3.9)	233 (3.9)	0.0	0.0	112 (2.0)	134 (2.2)	0.2	10.0
Florida	707 (3.6)	689 (3.5)	−0.1	−2.8	2,126 (11.0)	2,091 (10.7)	−0.3	−2.7
Hawaii	10 —^§§§^	—^§§§^	—^§§§^	—^§§§^	—^§§§^	—^§§§^	—^§§§^	—^§§§^
Indiana	327 (5.3)	311 (5.0)	−0.3	−5.7	649 (10.5)	713 (11.5)	1.0	9.5
Kentucky	269 (6.6)	140 (3.3)	−3.3**^†††^**	−50.0**^†††^**	780 (19.1)	744 (17.9)	−1.2	−6.3
Michigan	783 (8.2)	633 (6.5)	−1.7**^†††^**	−20.7**^†††^**	1,368 (14.4)	1,531 (16.0)	1.6**^†††^**	11.1**^†††^**
Minnesota	111 (2.0)	93 (1.7)	−0.3	−15.0	184 (3.5)	202 (3.7)	0.2	5.7
Mississippi	34 (1.3)	39 (1.4)	0.1	7.7	81 (2.9)	72 (2.6)	−0.3	−10.3
Texas	569 (2.0)	668 (2.3)	0.3**^†††^**	15.0**^†††^**	348 (1.2)	358 (1.2)	0.0	0.0

* Deaths were classified using *the International Classification of Diseases, Tenth Revision* (ICD–10). Drug overdose deaths were identified using underlying cause-of-death codes X40–X44, X60–X64, X85, and Y10–Y14. Rates are age-adjusted using the direct method and the 2000 U.S. standard population, except for age-specific crude rates. All rates were per 100,000 population.

^†^ Drug overdose deaths, as defined, that have heroin (T40.1) as a contributing cause.

^§^ Drug overdose deaths, as defined, that have semisynthetic opioids other than methadone (T40.4) as a contributing cause.

^¶^ Categories of deaths are not exclusive as deaths might involve more than one drug category. Summing of categories will result in more than the total number of deaths in a year.

** Data on Hispanic origin should be interpreted with caution; studies comparing Hispanic origin on death certificates and on Census surveys have shown inconsistent reporting on Hispanic ethnicity. Potential race misclassification might lead to underestimates for certain categories, primarily American Indian/Alaska Native non-Hispanic and Asian/Pacific Islander non-Hispanic decedents. https://www.cdc.gov/nchs/data/series/sr_02/sr02_172.pdf.

^††^ By the 2013 National Center for Health Statistics Urban-Rural Classification Scheme for Counties. https://www.cdc.gov/nchs/data_access/urban_rural.htm.

^§§^ *Northeast*: Connecticut, Maine, Massachusetts, New Hampshire, New Jersey, New York, Pennsylvania, Rhode Island, and Vermont. *Midwest*: Illinois, Indiana, Iowa, Kansas, Michigan, Minnesota, Missouri, Nebraska, North Dakota, Ohio, South Dakota, and Wisconsin. *South*: Alabama, Arkansas, Delaware, District of Columbia, Florida, Georgia, Kentucky, Louisiana, Maryland, Mississippi, North Carolina, Oklahoma, South Carolina, Tennessee, Texas, Virginia, and West Virginia. *West*: Alaska, Arizona, California, Colorado, Hawaii, Idaho, Montana, Nevada, New Mexico, Oregon, Utah, Washington, and Wyoming.

^¶¶^ Analyses were limited to states meeting the following criteria. States with very good to excellent reporting had ≥90% of drug overdose deaths mention at least one specific drug in 2017, with the change in drug overdose deaths mentioning of at least one specific drug differing by <10 percentage points from 2017 to 2018. States with good reporting had 80% to <90% of drug overdose deaths mention at least one specific drug in 2017, with the change in the percentage of drug overdose deaths mentioning at least one specific drug differing by <10 percentage points from 2017 to 2018. States included also were required to have stable rate estimates (i.e., based on ≥20 deaths in at least two of the following drug categories: opioids, prescription opioids, synthetic opioids other than methadone, and heroin).

*** Absolute rate change is the difference between 2017 and 2018 rates. Relative rate change is the absolute rate change divided by the 2017 rate, multiplied by 100. Nonoverlapping confidence intervals based on the gamma method were used if the number of deaths was <100 in 2017 or 2018, and z-tests were used if the number of deaths was ≥100 in both 2017 and 2018.

^†††^ Statistically significant (p-value <0.05).

^§§§^ Cells with nine or fewer deaths are not reported. Rates based on <20 deaths are not considered stable rate estimates and are not reported.

Death rates involving synthetic opioids increased from 9.0 per 100,000 population in 2017 to 9.9 in 2018 and accounted for 67.0% of opioid-involved deaths in 2018. These rates increased from 2017 to 2018 among males and females, persons aged ≥25 years, non-Hispanic whites, non-Hispanic blacks, Hispanics, non-Hispanic Asian/Pacific Islanders, and in large central metro, large fringe metro, medium metro, and small metro counties. Synthetic opioid–involved death rates increased in the Northeast, South and West and remained stable in the Midwest. Rates increased in 10 states and decreased in two states. The largest relative increase occurred in Arizona (92.5%), and the largest absolute increase occurred in Maryland and Missouri (4.4 per 100,000 in both states); the largest relative and absolute decrease was in Ohio (–20.7%, –6.7). The highest synthetic opioid–involved death rate in 2018 occurred in West Virginia (34.0 per 100,000).

## Discussion

During 1999–2018, opioids were involved in 446,032 deaths in the United States.^§§§^ From 2017 to 2018, relative decreases occurred in death rates involving all drug overdoses (–4.1%), all opioids (–2.0%), prescription opioids (–13.5%), and heroin (–4.1%); a relative increase occurred in the rate of overdose deaths involving synthetic opioids (10.0%). Decreases in all opioid-involved death rates were largely driven by those involving prescription opioids. The number of filled opioid prescriptions peaked in 2012 and decreased thereafter (*4*). Efforts to reduce high-dose opioid prescribing^¶¶¶^ (*4*) have increased and have contributed to decreases in prescription opioid–involved deaths. Factors that might be contributing to the decrease in heroin-involved deaths include fewer persons initiating heroin use (*5*), shifts from a heroin-based market to a fentanyl-based market (*6*), increased treatment provision for persons using heroin, and expansion of naloxone access (*5*,*7*). Increases in synthetic opioid–involved deaths are likely driven by proliferation of IMF or fentanyl analogs in the illicit drug supply (*3*,*5*,*6*). According to the Drug Enforcement Administration, fentanyl was the most identified synthetic opioid found during drug seizures in the first half of 2017 (*6*); in addition, fentanyl reports in all regions increased during 2014–2018.**** This is consistent with current findings indicating recent increases in synthetic opioid–involved death rates in all regions except the Midwest.

The findings in this report are subject to at least five limitations. First, postmortem toxicology testing varies by jurisdiction; improvements in testing might account for some reported increases. Second, the percentage of 2017 and 2018 death certificates with at least one drug specified varied among states and over time, limiting opioid subcategory rate comparisons. Third, because heroin is metabolized to morphine (*8*), some heroin deaths might have been misclassified as morphine deaths, resulting in an underreporting of heroin deaths. Fourth, potential race misclassification might have led to underestimates for certain categories, particularly American Indian/Alaska Natives and Asian/Pacific Islanders.^††††^ Finally, adequate drug specificity data were available from only 38 states and DC, which might limit generalizability of state-based analyses.

From 2017 to 2018, small decreases occurred in all overdose deaths and in deaths involving all opioids, prescription opioids, and heroin; however, deaths involving synthetic opioids continued to increase in 2018 and accounted for two thirds of opioid-involved deaths. Findings also highlight increases in deaths among non-Hispanic blacks and Hispanics, indicating the need for culturally tailored interventions that address social determinants of health and structural-level factors. In addition, changing substance use patterns, including the resurgence of methamphetamine use, particularly among persons using opioids (*9*) and the mixing of opioids with methamphetamine and cocaine in the illicit drug supply (*6*), have continued to make the drug overdose landscape more complicated and surveillance and prevention efforts more challenging. To sustain decreases and prevent continued increases, continued urgent action is needed. Overdose Data to Action^§§§§^ is a 3-year cooperative agreement through which CDC funds health departments in 47 states, DC, two territories, and 16 cities and counties for surveillance and prevention efforts. These measures include obtaining more timely data on all drug overdoses, improving toxicology to better identify polysubstance-involved deaths, enhancing linkage to treatment for persons with opioid use disorder and risk for opioid overdose, improving prescription drug monitoring programs, implementing health systems interventions, partnering with public safety, and implementing other innovative surveillance and prevention activities. Because of the reductions observed in deaths involving prescription opioids, continued efforts to encourage safe prescribing practices, such as following the CDC Guideline for Prescribing Opioids for Chronic Pain (*10*) might be enhanced by increased use of nonopioid and nonpharmacologic treatments for pain. Additional public health efforts to reduce opioid-involved overdose deaths include expanding the distribution of naloxone, addressing polysubstance use, and increasing the provision of medication-assisted treatment. Enhanced and coordinated multisectoral surveillance of the illicit drug supply^¶¶¶¶^ to track emerging threats, including the type and amount of specific drugs, could also help prevent overdoses. A comprehensive, multisectoral surveillance, prevention, and response approach remains critical for sustaining and expanding preliminary successes in reducing opioid-involved overdose deaths and specifically curtailing synthetic opioid–involved deaths and other emerging threats.

**Summary**

**What is already known about this topic?**

In 2017, 68% of the 70,237 U.S. drug overdose deaths involved an opioid. During 2016–2017, deaths involving all opioids and synthetic opioids increased; deaths involving prescription opioids and heroin remained stable.

**What is added by this report?**

Opioids were involved in approximately 70% (46,802) of drug overdose deaths during 2018, representing decreases from 2017 in overdose death rates involving all opioids (2% decline), prescription opioids (14%), and heroin (4%); rates involving synthetic opioids increased 10%.

**What are the implications for public health practice?**

Surveillance of overdose and polysubstance use trends and the illicit drug supply to track emerging threats, enhancing linkage to treatment, and a multisectoral response are critical to sustaining and accelerating declines in opioid-involved deaths.

## Footnotes

* Natural opioids include morphine and codeine. Semisynthetic opioids include oxycodone, hydrocodone, hydromorphone, and oxymorphone. Prescription opioids include methadone, natural, and semisynthetic opioids. Synthetic opioids include methadone, tramadol, and fentanyl (prescription and illicitly manufactured). Heroin is an illicit opioid made from morphine.

† https://www.cdc.gov/nchs/nvss/deaths.htm.

§ Drug overdose deaths, as defined, that have opium (T40.0), heroin (T40.1), natural and semisynthetic opioids (T40.2), methadone (T40.3), synthetic opioids other than methadone (T40.4) or other and unspecified narcotics (T40.6) as a contributing cause.

¶ For example, a death involving both heroin and a synthetic opioid other than methadone would be included in both the “heroin” and “synthetic opioid other than methadone” death rates.

** Age-adjusted death rates were calculated using the 2000 U.S. Census standard population age distribution https://www.cdc.gov/nchs/data/nvsr/nvsr61/nvsr61_04.pdf.

†† Categories were determined by the 2013 National Center for Health Statistics Urban-Rural Classification Scheme for Counties. *Large central metro:* counties in metropolitan statistical areas (MSAs) of ≥1 million population that 1) contain the entire population of largest principal city of the MSA; or 2) have their entire population contained in the largest principal city of the MSA; or 3) contain at least 250,000 inhabitants of any principal city of the MSA. *Large fringe metro:* counties in MSAs of ≥1 million population that did not qualify as large central metro counties. *Medium metro:* counties in MSAs of populations of 250,000–999,999. *Small metro:* counties in MSAs of populations less than 250,000. *Micropolitan (nonmetropolitan):* counties in micropolitan statistical areas. *Noncore (nonmetropolitan):* nonmetropolitan counties that did not qualify as micropolitan. https://www.cdc.gov/nchs/data_access/urban_rural.htm.

§§ *Northeast*: Connecticut, Maine, Massachusetts, New Hampshire, New Jersey, New York, Pennsylvania, Rhode Island, and Vermont. *Midwest*: Illinois, Indiana, Iowa, Kansas, Michigan, Minnesota, Missouri, Nebraska, North Dakota, Ohio, South Dakota, and Wisconsin. *South*: Alabama, Arkansas, Delaware, District of Columbia, Florida, Georgia, Kentucky, Louisiana, Maryland, Mississippi, North Carolina, Oklahoma, South Carolina, Tennessee, Texas, Virginia, and West Virginia. *West*: Alaska, Arizona, California, Colorado, Hawaii, Idaho, Montana, Nevada, New Mexico, Oregon, Utah, Washington, and Wyoming.

¶¶ Adequate drug specificity requires that at least one specific drug is named on the death certificate.

*** State-level analyses comparing death rates from 2017 to 2018 included 38 states and D.C. that met the following criteria: 1) >80% of drug overdose death certificates named at least one specific drug in 2017 and 2018; 2) change from 2017 to 2018 in the percentage of death certificates reporting at least one specific drug was <10 percentage points; and 3) ≥20 deaths occurred during 2017 and 2018 in at least two opioid subcategories examined. States whose reporting of any specific drug or drugs involved in an overdose changed by ≥10 percentage points from 2017 to 2018 were excluded because drug-specific overdose numbers and rates might have changed substantially from 2017 to 2018 as a result of changes in reporting.

††† Z-tests were used if the number of deaths was ≥100; a p-value of <0.05 was considered to be statistically significant. Nonoverlapping confidence intervals based on the gamma method were used if the number of deaths was <100 in 2017 or 2018. This method of comparing confidence intervals is a conservative method for estimating statistical significance; caution should be observed when interpreting a nonsignificant difference when the lower and upper limits being compared overlap only slightly. https://www.cdc.gov/nchs/data/nvsr/nvsr68/nvsr68_09-508.pdf.

§§§
https://wonder.cdc.gov .

¶¶¶ High-dose prescribing rates include prescriptions with daily dosage of ≥90 morphine milligram equivalents.

**** https://www.nflis.deadiversion.usdoj.gov/DesktopModules/ReportDownloads/Reports/NFLISDrug2018MY.pdf.

†††† Data for Hispanic origin should be interpreted with caution; studies comparing Hispanic origin on death certificates and on Census surveys have shown inconsistent reporting on Hispanic ethnicity. Potential race misclassification might lead to underestimates for certain categories, primarily American Indian/Alaska Native non-Hispanic and Asian/Pacific Islander non-Hispanic decedents. https://www.cdc.gov/nchs/data/series/sr_02/sr02_172.pdf.

§§§§ https://www.cdc.gov/drugoverdose/od2a/index.html.

¶¶¶¶ https://www.nflis.deadiversion.usdoj.gov/DesktopModules/ReportDownloads/Reports/NFLIS-Drug-AR2018.pdf.
